# Effect of sprinting velocity on anterior cruciate ligament and knee load during sidestep cutting

**DOI:** 10.3389/fbioe.2023.1033590

**Published:** 2023-02-07

**Authors:** Jeheon Moon, Dohoon Koo, Sungmin Kim, Siddhartha Bikram Panday

**Affiliations:** ^1^ Department of Physical Education, Korea National University of Education, Chungbuk, Republic of Korea; ^2^ Department of Exercise Prescription, Jeonju University, Chonbuk, Republic of Korea; ^3^ Institute of School Physical Education, Korea National University of Education, Chungbuk, Republic of Korea; ^4^ Department of Physical Education, Hanyang University, Seoul, Republic of Korea; ^5^ Department of Art and Sportainment, Hanyang University, Seoul, Republic of Korea

**Keywords:** musculoskeletal modeling, ACL injury, velocity, cutting, opensim

## Abstract

The purpose of the study was to investigate the effect of an increase in sprinting velocity on the anterior cruciate ligament (ACL) load, knee joint load, and activation of femoral muscles using the musculoskeletal modeling approach. Fourteen high school male athletes were recruited (age: 17.4 ± 0.7 years, height: 1.75 ± 0.04 m, weight: 73.3 ± 8.94 kg), with the right foot dominant and physical activity level of about 3–4 h per day. The kinematics, kinetics, and co-contraction index (CCI) of the extensors and flexors of the right leg’s femoral muscles were calculated. The anterior cruciate ligament load was estimated using the musculoskeletal modeling method. In the results, it was observed that the anterior cruciate ligament load (*p* < 0.017) increased as sidestep cutting velocity increased, resulting in increased adduction (*p* < 0.017) and the internal rotation moment of the knee joint. This was significantly higher than when sprinting at a similar velocity. The co-contraction index result, which represents the balanced activation of the femoral extensor and flexor muscles, showed a tendency of decrement with increasing sprinting velocity during sidestep cutting (*p* < 0.017), whereas no significant differences were observed when running at different sprinting conditions. Therefore, we postulate that factors such as knee joint shear force, extended landing posture with increasing sprinting velocity, internal rotation moment, and femoral muscle activity imbalance influence the increase of anterior cruciate ligament load during a sidestep cutting maneuver.

## 1 Introduction

Anterior Cruciate Ligament (ACL) injuries frequently occur when the knee joint is subjected to high loading during a sudden change in the whole body velocity (Pflum et al., 2004; Kernozek and Ragan 2008; O’Connor et al., 2009; Laughlin et al., 2011) such as a stop jump (Lin et al., 2009) or sidestep cutting (McLean et al., 2004; Pollard et al., 2005; McLean et al., 2008). Various biomechanical approaches have been employed to speculate on the mechanism of ACL injury by quantifying changes in kinematics, kinetics, and muscle activities (Weinhandl et al., 2010; Weinhandl et al., 2011). Studies report ACL injuries occur when landing with the knee elongated in the abduction position (Olsen et al., 2004; Krosshaug et al., 2005). Particularly, knee joint angles in the range of 9°∼27° while impacting the ground after jumping and the height of the knee were reported to significantly increase the risk of an ACL injury (Olsen et al., 2005). Moreover, changes in shear force and internal rotation of the knee joint have a greater effect on ACL load than the external rotation of the knee (Markolf et al., 1995).

The involvement of femoral muscle activation should be emphasized to better understand the ACL injury mechanism. When both agonistic and antagonistic muscles contract simultaneously, this is known as the “co-contraction of muscles” (Kellis et al., 2003). Hurd and Snyder-Mackler (2007) observed that high antagonistic muscle co-contraction can exhibit a knee stiffening strategy and improve the stability of the knee joint. The strong quadriceps femoris contraction increases ACL load and also correlates with ground reaction force (GRF). Further, when the knee is extended, the coupled moment acts on the posterior joint, increasing the risk of ACL injury (Fleming et al., 2001; DeMorat et al., 2004). Boden et al. (2000) reported that co-contraction of the quadriceps femoris and the biceps femoris (BF) decreases ACL strain. Khandha et al. (2019), on the contrary, argued that prolonged high muscle co-contraction may result in high joint loads, adversely affecting knee cartilage integrity. Additionally, several studies indicate that pre-activation of the quadriceps femoris before landing and co-contraction of the quadriceps femoris with the biceps femoris may aid in preventing the ACL load from increasing (Hewett et al., 2005; Kernozek and Ragan 2008; Hashemi et al., 2010).

Studies that investigated the factors contributing to ACL injuries caused during the sidestep cutting maneuver were conducted at a constant sprinting velocity (McLean et al., 2004; O’Connor et al., 2009; Weinhandl et al., 2013). ACL loading has been reported to occur as a result of a complex interaction between sagittal plane shear forces (i.e., quadriceps, hamstrings, and tibiofemoral) and frontal and transverse plane knee moments (Bedo et al., 2021; Weinhandl et al., 2013). Importantly, the findings of previous studies suggest that unanticipated movements such as sidestep cutting increase anterior cruciate ligament loading. Although the majority of non-contact ACL injuries in sporting scenarios are caused by excessive pressure or load on the knee joint during the weight-shifting process, little attention has been paid to the effect of running velocity and weight-shifting situations (i.e., sidestep cutting) on ACL injuries. According to Olsen et al. (2004), when running at a high velocity (5–8 m/s), increased ACL loading in the sagittal plane increases the vulnerability of injury. The increased ACL load was attributed to increased peak posterior GRF, knee extension moment, and stiffness, as well as decreased knee flexion range of motion (Yu and Garrett 2007; Lin et al., 2009; Taylor et al., 2011; Dai et al., 2012; Taylor et al., 2013).

Therefore, the purpose of the study was to investigate whether increasing sprinting velocity (3 m/s, 4 m/s, or 5 m/s) has an influence on ACL load, knee joint load, and femoral muscle co-activation using a musculoskeletal modeling technique. We hypothesize that 1) an increase in running velocity will increase the knee joint angle in all three directions, 2) an increase in running velocity will increase ACL load, and 3) an increase in velocity will decrease the co-contraction index.

## 2 Materials and methods

### 2.1 Participants

The study included fourteen high school male athletes (age: 17.4 ± 0.7 years, height: 1.75 ± 0.04 m, weight: 73.3 ± 8.94 kg). All of the individuals were right-foot dominant and engaged in varied sports activities for an average of 3–4 h per day. None of the subjects had a history of morbidity or musculoskeletal injury within the preceding 6 months. All individuals volunteered to participate in the experiment and gave their consent after reading the research instructions and signed the consent forms. The Institutional Review Board of Seoul National University (IRB Number: 1511/022-001) approved the research to ensure that it adhered to the Declaration of Helsinki’s ethical principles (1975, revised 1983).

### 2.2 Apparatus

Nineteen infrared cameras (Oqus 7+, Gothenburg, Sweden) and a single force plate (Kistler Type 9287BA, Winterthur, Switzerland) were used to measure the motion data and the GRF. The muscle activities were measured using a wireless electromyography (EMG) system (Noraxon DTS, Scottsdale, AZ, United States). The three measurement systems were synchronized, and the set of synchronized data was sent to the main computer. The sampling rates were set at 200 Hz for the motion data, GRF at 2,000 Hz, and EMG at 1,500 Hz.

### 2.3 Experimental procedures

The participants were provided with a set of experimental attire and shoes prepared by the experimenter. Before the main experiment, the participants performed warm-up exercises for about 15 min. Thereafter, their skin was shaved with an alcohol pad to clean the skin and reduce the skin’s impedance. Disposable dual-electrodes (Noraxon TM, Scottsdale, Arizona) were also attached to the belly of five muscle sites (Hermens et al., 1999) in the right lower extremities, including the rectus femoris, vastus medialis, and vastus lateralis, as well as flexors such as the biceps femoris and semitendinosus. The electrodes were further secured with adhesive cotton tape to minimize motion artifacts during the tasks. We then measured the manual isometric maximum voluntary contraction (MVC) test for extensors and flexors separately with the method described by Konrad (Konrad 2005). For the MVC test of the rectus femoris, vastus medialis, and vastus lateralis, participants were instructed to do a single-leg knee extension from a seated posture with the leg strapped to the chair and the knee flexed from 90° to 70°. For the MVC flexor test, participants lay in a prone position with their hips firmly fastened with a belt and conducted a unilateral knee flexion at 20–30°. In both tests, participants were asked to slowly increase the force until they reached their maximum after 3–5 s, hold for at least 5 s, and relax promptly. Throughout the MVC test, the experimenter verbally encouraged the participants to perform at their maximum. For each muscle group, the MVC test was repeated three times, and a 1-min rest was given after each repetition (Rutherford et al., 2011). The MVC values of each muscle site from three trials were averaged and later used for EMG normalization. After the MVC tests, participants were given 10–15 min for recovery. We then attached spherical passive reflective markers (12.7 mm in diameter) at the anatomically bony landmarks to define joints and segments (Cappozzo et al., 1997; Wu et al., 2002; Wu et al., 2005; Collins et al., 2009). A 13-segment kinematic model was employed, which consisted of the head, trunk, pelvis, and bilateral upper arms, lower arms, thighs, shanks, and feet.

**FIGURE 1:**
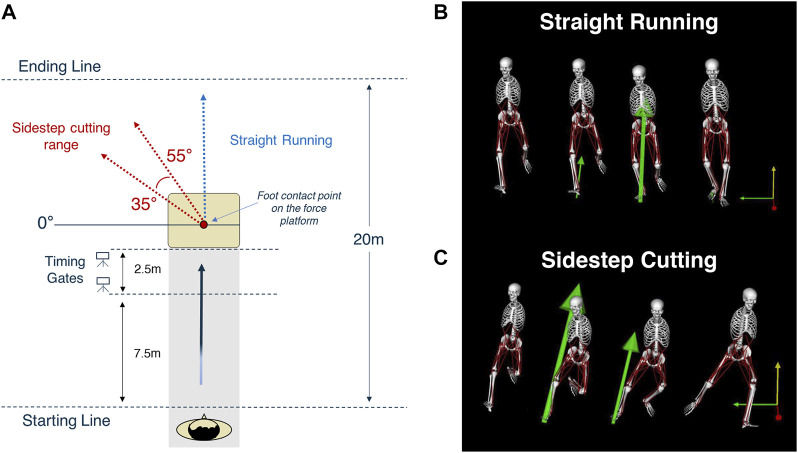
Experimental Procedure. **(A)** The sidestep cutting technique was carried out between 35° and 55° to the left of the path of travel. Sprinting velocity was controlled by monitoring the participant’s velocity in the 2.5 m range prior to contact with the ground reaction force measurement apparatus. **(B,C)** The two running condition types performed in the study. The image were extracted at using open source model provided in OpenSim (Stanford University, Stanford, CA, United States).

The main experiment included two sprinting *TYPES*, i.e., sidestep cutting and straight running at three constrained sprinting velocities (3 m/s, 4 m/s, and 5 m/s) (McLean et al., 2004; Weinhandl et al., 2013; Xu et al., 2018). We monitored the participants’ sprinting velocity using two timing gates (Witty Microgate, Bolzano, Italy), which measure the average passage time. The timing gates were installed at intervals of 2.5 m before the force plate (Figure 1). Considering the variability in performance, we collected data for only successful trials that came within ±5% for each velocity condition. Participants in both the sidestep cutting and straight running conditions ran a total of 10 m and were required to plant their right foot on the force plate. They were asked to maintain a constant velocity throughout the run and were given enough time for practice runs for each condition and type. Before the initiation of trials, the participants were notified of the types and velocities required for each trial. For the sidestep cutting condition, the participants were instructed to change their running direction to the left after planting on the force plate with the right leg. The range of sidestep cutting was set at 35°–55° to the left of the proceeding direction (Donnelly et al., 2012; McLean et al., 2004; McLean et al., 2008; Weinhandl et al., 2013). For the straight running condition, the participants were instructed to plant their right foot at the same location and run straight forward without any deceleration (Weinhandl et al., 2013). The order of conditions was randomized using MATLAB’s random number generator function (version 2016b, MathWorks, Inc., United States). Each participant performed five trials for each condition, and a 3-min rest was given between each trial. Thus, a total of 30 trials were collected (5 trials × 3 velocities × 2 running types).

### 2.4 Data analysis

The measured data was labeled and extracted to a C3D file for further processing with Visual3D software. For kinematics data, spline interpolation up to 10 frames and a Butterworth 4th order low-pass filter at a cut-off frequency of 15 Hz was applied (Donnelly et al., 2012; Mudie et al., 2017). The kinetics data were filtered using a Butterworth 4th-order low-pass filter at a cut-off frequency of 20 Hz (Weinhandl et al., 2013). Similarly, raw EMG signals were filtered using a Butterworth 4th-order high-pass filter at a cut-off frequency of 20 Hz (De Luca et al., 2010). This was followed by full-wave rectification and re-filtering conducted with a Butterworth 4th-order low-pass filter at a cut-off frequency of 15 Hz (Laughlin et al., 2011).

#### 2.4.1 Kinematic and kinetic data analysis

The femoral epicondyle markers (Fonseca et al., 2020) and the malleoli markers (Wu et al., 2002) were used to define the knee and ankle joint centers, respectively. The three-dimensional knee joint angle was calculated using an inverse kinematics algorithm. Negative values for flexion, abduction and external rotation were used to define the knee joint angles. The knee joint force and moment were calculated using an inverse dynamics algorithm. Positive values for extension, adduction and internal rotation were used to define the knee joint moments. The shear force that acts in the anterior-posterior and medial-lateral directions was referred to as the knee joint force. Peak values for different parameters were calculated at the instant of planting for each trial, which was identified by a force profile in the vertical direction (Fz). For interparticipant comparability, body mass was used to normalize the shear force and body mass and height to normalize the knee joint moment.

#### 2.4.2 ACL modeling

The ACL force was calculated using the OpenSim musculoskeletal simulation software (version 3.3, Stanford University, Stanford, CA, United States). We employed the gait2354 model, which has twelve segments with twenty-three degrees of freedom, including the lower leg and trunk (DOF). Fifty-four muscles were included, but only one DOF (flexion/extension) for the knee joint (Delp et al., 1990; Anderson and Pandy 1999). The ACL load was calculated using a model that took into account the knee joint’s flexion/extension, adduction/abduction, internal/external rotation, and the maximum isometric force of the muscles acting around the knee joint. As a result, the improved model was a 3-DOF ball-and-socket joint with flexion/extension ranges of −120 to +10°, adduction/abduction ranges of +15 to −15°, and internal/external rotation ranges of +30 to −45° (Kar and Quesada 2012; Kar and Quesada 2013). Previous studies were used to obtain detailed ACL parameters such as location, length, cross-section, and strain (Cohen et al., 2009; Kar and Quesada 2012; Kar and Quesada 2013).

The inverse kinematics and dynamics calculations were performed after the participants’ static standing information data was scaled on the modified ACL model. After reproducing the measured motion using the residual reduction algorithm, the computed muscle control (CMC) was used to construct a set of muscle excitations (Delp et al., 1990; Thelen et al., 2003; Thelen and Anderson 2006). Finally, the ACL force was computed using Hill’s model, which may be found in the formula below (Hill 1938; Kar and Quesada 2012; Kar and Quesada 2013).

$$f_{m}^{*}=\left[a_{m}^{*}f_{lv}\left(l_{m}^{*},i_{m}^{*}\right)+f_{psv}\left(l_{m}^{*}\right)\right]\cos\left(\alpha_{m}^{*}\right)$$

Here, $a_{m}^{*}$ refers to muscle activity, $f_{lv}$ refers to the active force exerted by the force-length-velocity curve based on Hill’s model, $l_{m}^{*}$ is the muscle’s length, $i_{m}^{*}$ refers to the velocity of the tendon acting in the muscle direction, $f_{psv}$ is the passive force, $a_{m}^{*}$ is the pennation angle of the muscle. The model was validated using a standard protocol, for which the residual force values of the RRA and CMC were considered. The outputs were compared to the threshold values presented in the OpenSim software used to evaluate simulations of walking and running (Lee et al., 2020).

#### 2.4.3 Co-contraction index of electromyography

The co-contraction index (CCI) was calculated for the femoral extensors (rectus femoris, vastus medialis, vastus lateralis) and flexors (biceps femoris, semitendinosus) muscles following normalization with the MVC test. The analytical duration was set to 100 milliseconds before and following the GRF peak. All calculations were performed using Matlab (R2009b, MathWorks, Inc., Natick, MA, United States), and the following formula was used to perform the computations (Park et al., 2011).

$$CCI=1-\frac{\left|M_{Extensor}\right|-\left|M_{Flexor}\right|}{\left|M_{Extensor}\right|+\left|F_{Flexor}\right|}=2\frac{\left|M_{Flexor}\right|}{\left|M_{Extensor}\right|+\left|M_{Flexor}\right|}$$

### 2.5 Statistical analysis

Repeated-measured ANOVAs were used to explore the effect of the main outcome variables by the two factors, *VELOCITY* (3 levels: 3, 4, and 5 m/s) and *TYPE* (2 levels: straight-run and cutting-run). Mauchly’s sphericity test was employed to confirm or reject the assumptions of sphericity. The Greenhouse-Geisser corrections were used when the sphericity assumption was rejected. The statistical power for all comparisons was computed, and for all planned comparisons, the power was over 0.7 from the pool of fourteen participants. For *post-hoc* comparison, paired *t*-tests were performed to explore further significant effects of three levels of velocity with Bonferroni *p*-value adjustments for multiple comparisons (*p* < 0.0083 instead of the nominal *p* < 0.05). For all statistical tests, the level of significance was set at *p* 0.05.

## 3 Results

### 3.1 Knee joint angle

**FIGURE 2:**
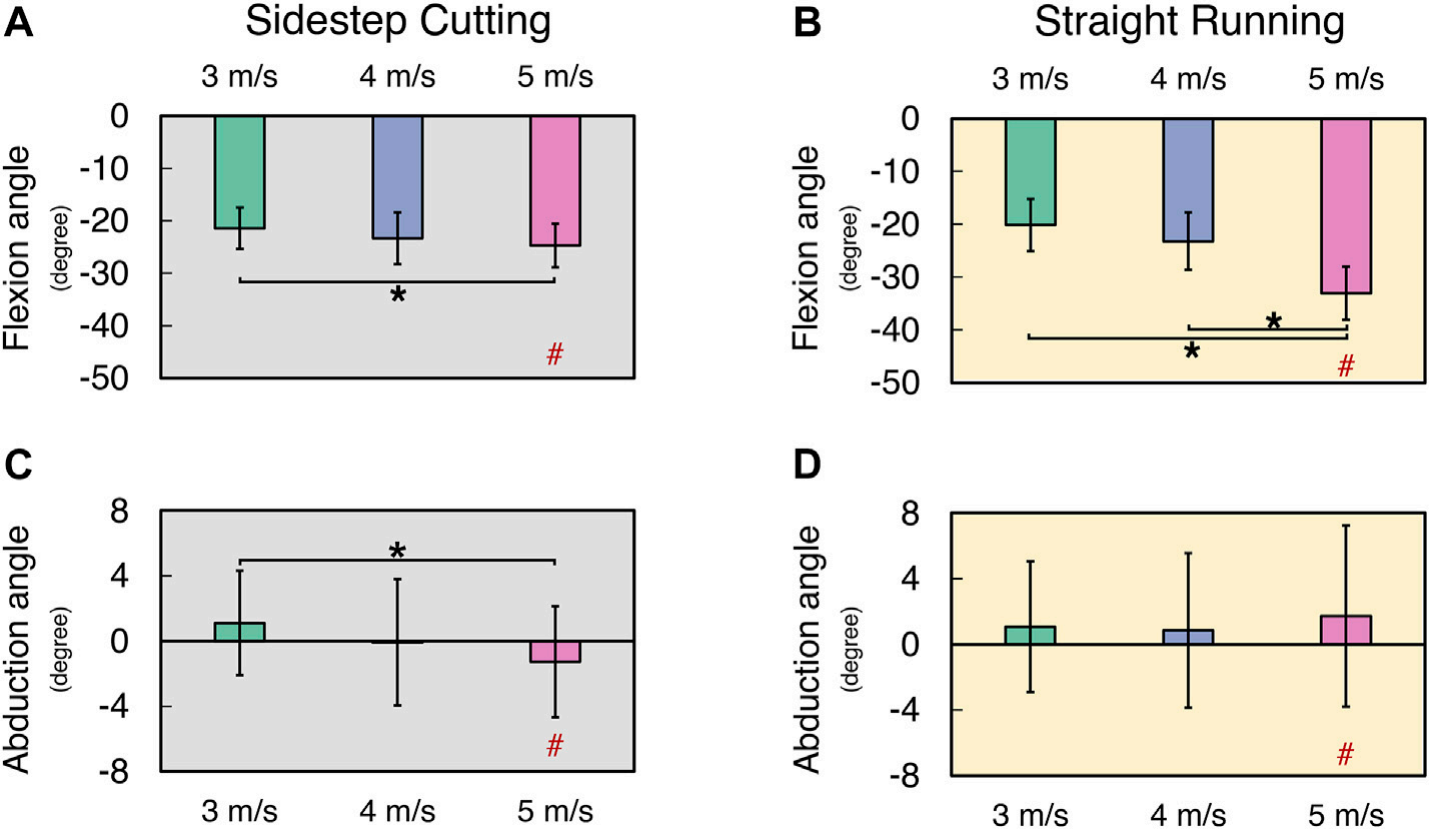
The mean and standard deviation of calculated variables during sidestep cutting (*Gray*) and straight running (*Yellow*). **(A,B)** Peak knee flexion angle, **(C,D)** Peak knee abduction angle. Note: Units expressed in degrees. Knee extension, adduction, and internal rotation are positive. Significant differences between *VELOCITY* denoted by asterisks (*) (*p* < 0.05). Significant differences between running *TYPES* for each condition denoted by hash (#) (*p* < 0.05).

In general, the knee flexion angles and knee abduction angles increased with running velocity, while there was no clear effect of the running velocity on the maximal internal rotation of the knee joint (Figure 2) (See Supplementary Files for details). For the knee flexion angle and knee abduction angle, the significant difference between the running types (e.g., straight and sidestep cutting runs) was only observed at the highest velocity (i.e., 5 m/s), which showed a larger knee flexion angle during straight running than during side step cutting. There was no significant difference in the knee internal rotation angle between the two running types. These findings were supported by a two-way repeated measure ANOVA separately on knee flexion angle, knee abduction angle, and knee internal rotation angle with factors *TYPE* (two levels: sidestep cutting and straight running) and *VELOCITY* (three levels: 3, 4, and 5 m/s). The main effects of *VELOCITY* (*F*_[2, 24]_ = 31.46, *p* < 0.001) and *TYPE* (*F*_[1, 12]_ = 5.49, *p* < 0.05) were significant on knee flexion angle only. The factor interaction *TYPE* × *VELOCITY* was significant on both the knee flexion angle and the knee abduction angle, which reflected the fact that a significant effect of *TYPE* on both the knee flexion angle and the knee abduction angle was observed only at the 5 m/s condition (*p* < 0.001 for flexion angle, *p* < 0.01 for abduction angle). A *post-hoc* comparison on knee flexion angle showed that knee flexion angle of 3 m/s < 5 m/s during the sidestep cutting condition (*p* < 0.017), and a knee flexion angle of 3 and 4 m/s < 5 m/s during the straight running condition (*p* < 0.017). Similarly, *post-hoc* comparison on knee abduction angle only showed a knee abduction angle of 3 m/s < 5 m/s during the sidestep cutting condition (*p* < 0.017), which confirmed a significant factor interaction on knee flexion angle and knee abduction angle.

### 3.2 ACL force and knee shear force

**FIGURE 3:**
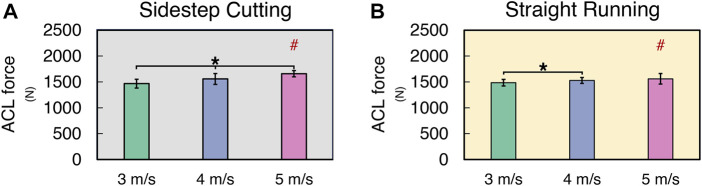
The mean and standard deviation of ACL force during **(A)** sidestep cutting (*Gray*) and **(B)** straight running (*Yellow*). Note: Units expressed in newton. Significant differences between *VELOCITIES* denoted by asterisks (*) (*p* < 0.05). Significant differences between running *TYPES* for each condition denoted by hash (#) (*p* < 0.05).

The ACL force normalized by the body mass (ACLF_NORM_) increased with running velocity in both sidestep cutting and straight running conditions, and ACLF_NORM_ in sidestep cutting and straight running conditions was similar in magnitude for 3 and 4 m/s velocity conditions (Figure 3) (See Supplementary Files for details). These findings were supported by the results of a two-way repeated measures ANOVA, which confirmed a significant main effect of *VELOCITY* on ACLF_NORM_ (*F*_[2, 24]_ = 14.95, *p* < 0.001) with a significant factor interaction (*F*_[2, 24]_ = 9.95, *p* < 0.01). Post-hoc comparisons showed ACLF_NORM_ at 3 m/s < 4 m/s < 5 m/s for the sidestep cutting condition (*p* < 0.017), and 3 m/s < 4 m/s and 5 m/s for the straight running condition (*p* < 0.017), which confirmed a significant factor interaction.

**FIGURE 4:**
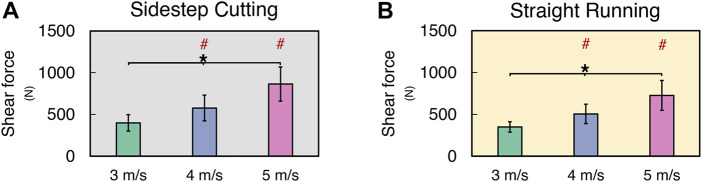
The mean and standard deviation of knee shear force during **(A)** sidestep cutting (*Gray*) and **(B)** straight running (*Yellow*). Note: Units expressed in newton. Significant differences between *VELOCITIES* denoted by asterisks (*) (*p* < 0.05). Significant differences between running *TYPES* for each condition denoted by hash (#) (*p* < 0.05).

The knee shear force (KSF) normalized by the body mass of individual subjects (KSF_NORM_) increased with running velocity in both sidestep cutting and straight running conditions (Figure 4) (See Supplementary Files for details). Also, KSF_NORM_ at 4 and 5 m/s velocities were larger in the sidestep cutting condition than in the straight running condition. A two-way repeated measures ANOVA with factor *TYPE* and *VELOCITY* confirmed the significant main effect of *VELOCITY* (*F*_[2, 24]_ = 76.46, *p* < 0.001) with a significant factor interaction (*F*_[2, 24]_ = 6.68, *p* < 0.01). The *post-hoc* comparison showed KSF_NORM_ in sidestep cutting > straight running at 4 and 5 m/s conditions (*p* < 0.017), not at 3 m/s conditions.

### 3.3 Knee joint moment

**FIGURE 5:**
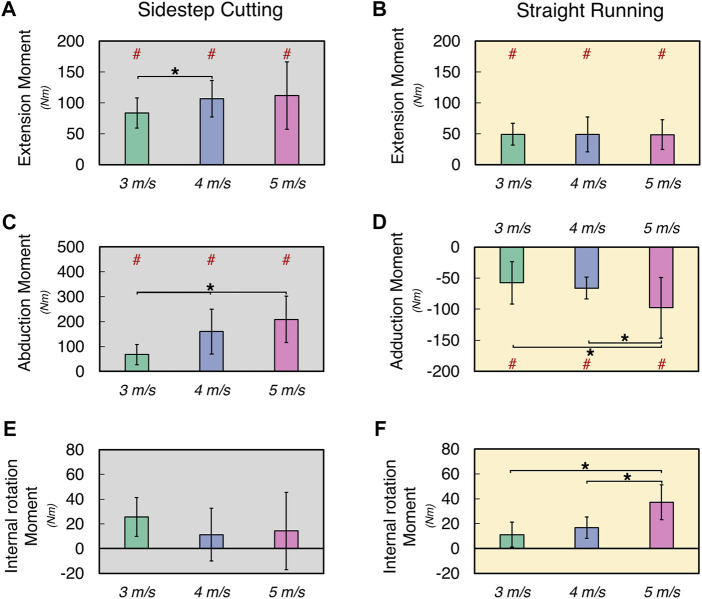
The mean and standard deviation of calculated variables during sidestep cutting (*Gray*) and straight running (*Yellow*). **(A,B)** Peak knee extension moment, **(C)** Peak knee abduction moment, **(D)** Peak knee adduction movement, **(E,F)** Internal rotation moment. Note: Units expressed in Newton-meters (Nm). Knee extension, adduction, and internal rotation are positive. Significant differences between *VELOCITY* denoted by asterisks (*) (*p* < 0.05). Significant differences between running *TYPES* for each condition denoted by hash (#) (*p* < 0.05).

The normalized knee extension moment (KEM_NORM_) increased only at medium velocity (i.e., 4 m/s) in the sidestep cutting condition, while no differences were observed in the straight running condition (Figures 5A, B) (See Supplementary Files for details). A two-way repeated measure ANOVA with factor *TYPE* and *VELOCITY* confirmed the significant main effect of *TYPE* (*F*_[1, 12]_ = 44.83, *p* < 0.001). The *post-hoc* comparison showed KEM_NORM_ at 3 m/s < 4 m/s for the sidestep cutting condition (*p* < 0.017), and KEM_NORM_ in sidestep cutting > straight running at 3, 4, and 5 m/s conditions (*p* < 0.017).

The normalized knee adduction moment (KADM_NORM_) increased with running velocity in the sidestep cutting condition, while the normalized knee abduction moment (KABM_NORM_) increased only at the highest velocity (i.e., 5 m/s) in the straight running (Figures 5C, D) (See Supplementary Files for details). These findings were supported by the results of a two-way repeated measures ANOVA, which confirmed the significant main effects of *TYPE* on KADM_NORM_ (*F*_[1, 12]_ = 168.76, *p* < 0.001) and *VELOCITY* on KADM_NORM_ (*F*_[2, 24]_ = 18.97, *p* < 0.001) with a significant factor interaction (*F*_[2, 24]_ = 38.89, *p* < 0.001). The *post-hoc* comparison showed KADM_NORM_ at 3 m/s < 4 m/s < 5 m/s for the sidestep cutting condition (*p* < 0.017), and KABM_NORM_ at 3 and 4 m/s < 5 m/s for the straight running condition (*p* < 0.017), which confirmed a significant factor interaction.

In the straight running condition, the normalized internal rotation moment (KIRM_NORM_) increased with running velocity, while no clear effect of running velocity was observed for KIRM_NORM_ in the sidestep cutting condition (Figures 5E, F) (See Supplementary Files for details). The results of the two-way repeated measure ANOVA revealed that the main effects of *VELOCITY* and *TYPE* had no statistical significance, but the factor interaction was significant on KIRM_NORM_ (*F*_[2, 24]_ = 21.28, *p* < 0.001). Post-hoc comparison only showed KIRM_NORM_ at 3 and 4 m/s < 5 m/s for the straight running condition (*p* < 0.017).

### 3.4 Co-contraction index (CCI)

**FIGURE 6:**
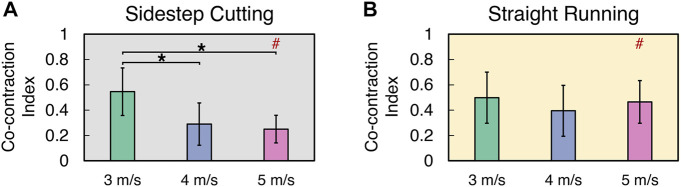
The mean and standard deviation of co-contraction index during **(A)** sidestep cutting (*Gray*) and **(B)** straight running (*Yellow*). Note: Significant differences between *VELOCITIES* denoted by asterisks (*) (*p* < 0.05). Significant differences between running *TYPES* for each condition denoted by hash (#) (*p* < 0.05).

The CCI of the flexors and extensors of femoral muscles decreased with running velocity during the sidestep cutting, while no clear effect of running velocity was observed in the straight running (Figure 6) (See Supplementary Files for details). Also, CCI at 4 and 5 m/s velocity were larger in the sidestep cutting condition than in the straight running condition. A two-way repeated measures ANOVA with factor *TYPE* and *VELOCITY* confirmed the significant main effects of *TYPE* (*F*_[1, 12]_ = 10.24, *p* < 0.01) and *VELOCITY* (*F*_[2, 24]_ = 14.85, *p* < 0.001) with a significant factor interaction (*F*_[2, 24]_ = 19.86, *p* < 0.001). Post-hoc comparisons showed CCI at 3 m/s < 4 and 5 m/s in the sidestep cutting condition (*p* < 0.017), while CCI during sidestep cutting < straight running and statistical significance was observed only in the 5 m/s condition (*p* < 0.017), but not at 3 and 5 m/s.

## 4 Discussion

The purpose of the study was to investigate the effect of increasing sprinting velocity on ACL load, knee kinematics, and CCI, for which we employed a musculoskeletal modeling approach. Our findings indicated that an increase in running velocity during the sidestep cutting condition increased knee abduction angles, while the increase in velocity during straight running increased knee flexion angles. In line with our initial hypothesis, the ACLF_NORM_, KSF_NORM_, KEM_NORM_, KADM_NORM_, and CCI increased with running velocity, as predicted, and had higher values in the sidestep cutting condition than in the straight running condition. KIRM_NORM_, on the other hand, showed no differences in running velocity or type.

### 4.1 Mechanism of changes in ACL loading with running velocities and types

The amplitude of ACL load is known to be influenced by knee flexion, with the maximum load found when the knee is flexed beyond 30° (DeMorat et al., 2004; Gabriel et al., 2004). The cadaveric investigation by Gabriel et al. (2004) confirmed this, revealing that the peak magnitude of ACL load was around 120 N at 15°–20° of knee flexion. The angle of knee flexion at the maximum magnitude of ACL load was also in the same range as the results of the current study. The angle of knee flexion increased with increasing running velocity, which coincided with an increase in ACL loading force during both the sidestep cutting and straight running. The highest ACL loading force was detected during the cutting run (5 m/s), although a considerably greater degree of knee flexion was observed during the straight running, which had a lower ACL loading force than the sidestep cutting. This indicates that knee flexion is not the only element to consider when estimating the risk of ACL injury (i.e., the ACL loading force). Running with cutting (rapid directional shift) at a faster velocity is likely to be more damaging to ACL damage, as shown by a greater loading force. The observed findings are consistent with the general assumption of ACL injury in terms of the level of ACL loading as a function of running velocity and type. Aside from knee flexion kinematics, the knee abduction/adduction kinematics differed across the two running types used in this study. The knee was adducted during straight running and abducted as the velocity increased. Even during the sidestep cutting, the knee was adducted at a slower pace.

In the study conducted by Markolf et al. (1995) applied a 100 N shear force to the tibia while varying the knee flexion (−)/extension (+) angles between −10° and 90°. The study found that knee abduction and internal rotation moments were the primary factors contributing to the increase in ACL load. While another female study found a knee abduction angle of 15°–20° during the sidestep cutting and landing (McLean et al., 2004; Olsen et al., 2004). When abrupt directional changes or deceleration were undertaken, analysis of real video from matches revealed external to internal tibial rotation (Boden et al., 2000). This type of situation can be referred to as movement behavior that frequently occurs with athletes during competitive matches. However, individuals in our study were able to predict the direction of the sidestep cutting in advance, which resulted in the direction switching being carried out in a state with the knee internal rotation angle.

### 4.2 Effect of running velocity and type on ACL load

Using our musculoskeletal model, we demonstrated the effect of running *VELOCITY* (i.e., the greater the running velocity, the greater the ACL loading force) and *TYPE* on the estimated maximal ACL load during the sidestep cutting. Previous studies have indicated a maximal ACL load of 681 N during sidestep cutting at a running velocity of 4.5–5.0 m/s, which is similar to our findings (Kernozek and Ragan 2008; Weinhandl et al., 2013). In comparison to our investigation, the result of the knee joint moment from Weinhandl et al., (2013) was significantly lower. Although the movement differed, when executing a double leg landing task at a height of 50 cm, the ACL loading was reported to be 1,150 N for the right and 1,136 N for the left in a study by Kar and Quesada (2012), who used a similar biomechanical model as our work. Additionally, the ACL load estimated using this musculoskeletal model is more likely to be true, given that the ACL injury threshold is approximately 2,000 N (Stapleton et al., 1998).

Previously, research indicated that the ACL load was influenced by the knee shear force acting on the knee adduction and internal moments as well as the contraction of the quadriceps femoris (Markolfet al., 1995; Fukuda et al., 2003; DeMorat et al., 2004). However, there are conflicting perspectives regarding the effect of the external rotation moment on the rise in ACL load (Markolfet al., 1995; Fleming et al., 2001). Similarly, several investigations have indicated that when a force of 2,000 N or greater is applied, the shear force causes an ACL injury (Shin et al., 2007; McLean et al., 2008). The knee shear force at 5 m/s was the greatest in our study, at 864.2 ± 204.7 N (1.156 ± 0.241 N/BW) in the sidestep cutting condition, but it was much lower than the 2,000 N reported in other investigations. Furthermore, our findings are consistent with the findings of McLean et al., 2004, who concluded that the shear force acting on the knee joint cannot cause the injury on its own.

The adduction moment of the knee joint has been implicated in prior research as a factor impacting ACL loading (Markolfet al., 1995; Fukuda et al., 2003; McLean et al., 2004; McLean et al., 2008). Additionally, when executing a sidestep cutting, the adduction moment of the knee was greater than the abduction moment (Weinhandl et al., 2013). Furthermore, previous research has indicated that the internal rotation moment of the knee joint is a significant factor in increasing ACL load (Markolfet al., 1995; Shin et al., 2009). Markolfet al, (1995) used a shear force of 100 N and a moment of 10 Nm at a constant knee angle (flexion from 5° to 90°) to observe variations in ACL stress. As a result, the ACL load of shear force and internal rotation moment was around 180 N when the knee joint was flexed at 15°, which is nearly 2.6 times higher than a load of shear force and external rotation moment of 70 N. However, in this investigation, the internal rotation moment at the ACL maximum load was unaffected by increasing running velocity during sidestep cutting but was greatest at 5 m/s during straight running. These findings suggest that the task performed in this study can be predicted in advance, thereby shortening the time spent in the transverse plane (Weinhandl et al., 2013). Additionally, further research is required to determine the degree of injury threshold for internal rotation moments (McLean et al., 2004).

### 4.3 Effect of running velocity and type on the femoral muscle

Another factor that has been shown to affect ACL load around knee joints is quadriceps and biceps femoris muscle contractions. Previous research has found that excessive quadriceps femoris muscle contraction causes forward displacement of the tibia, which increases ACL load (Markolfet al., 1995; Fleming et al., 2001; DeMorat et al., 2004). Furthermore, it has been reported that if the femoral muscles are imbalanced, they become less mobilized or fatigued, causing a delay in muscle contraction time, which increases the ACL load (Boden et al., 2000; Hewett et al., 2005). However, Simonsen et al. (2000) hypothesized that the biceps femoris would have a negligible effect on ACL load reduction during the sidestep cutting since the biceps femoris has a low level of muscle activity (34%–39% of MVC). Additionally, the CCI of the extensor and flexor muscles during the sidestep cutting at 5 m/s was 0.244 ± 0.107%, which was found to be statistically less than the CCI in the 3 m/s condition. When the running velocity was increased, the ratio of muscular activity in the rectus femoris, vastus medialis, and vastus lateralis (extensor muscles) increased. While the flexor muscle’s biceps femoris and semitendinosus muscular activation ratios were observed to be relatively lower and are considered to increase the risk of injury.

## 5 Conclusion

The goal of this study was to determine the effect of increasing sprinting velocity on ACL load, knee kinematics, and CCI, which was achieved using musculoskeletal modeling. As a result, the shear force, extended landing posture with increased running velocity, and femoral muscle imbalance activity all influenced the ACL load during a sidestep cutting. We suggest that to compensate for muscular imbalances in the lower limb and the increased load associated with increasing sidestep cutting velocity, it would be beneficial to strengthen the hamstring muscles and concentrate on knee flexion during landing.

## Data Availability

The raw data supporting the conclusion of this article will be made available by the authors, without undue reservation.
