# Supplementary material for: Effect of sprinting velocity on anterior cruciate ligament and knee load during sidestep cutting
Source: Front Bioeng Biotechnol. 2023 Feb 7;11:1033590. doi: 10.3389/fbioe.2023.1033590 (PMC9941960; doi:10.3389/fbioe.2023.1033590)
Supplement: Supplementary file 1 [file Table1.DOCX]

Supplementary Material

# Supplementary Table

Table 1: Results of Two-way repeated ANOVA for knee joint, ACL ligament ACL and muscle co-contraction index variables during the landing phase of sidestep cutting maneuver (n=14).

| **Variables** | **Cutting** | | |  | **Running** | | |  |
| --- | --- | --- | --- | --- | --- | --- | --- | --- |
|  | **3 m/s** | **4 m/s** | **5 m/s** | **Effect size** | **3 m/s** | **4 m/s** | **5 m/s** | **Effect size** |
| **Knee angle (°)** |  |  |  |  |  |  |  |  |
| Sagittal* | -21.44±3.93 | -23.35±4.93 | -24.73±4.17^ac^ | .093 | -20.14±4.94 | -23.21±5.43 | -33.03±5.02^ab^ | .554 |
| Frontal* | 1.095±3.200 | -0.085±3.878 | -1.277±3.400^ac^ | .076 | 1.073±3.971 | 0.849±4.713 | 1.714±5.515 | .006 |
| Transverse | 2.006±5.629 | 3.269±5.905 | 2.787±6.126 | .008 | -0.425±4.236 | 2.135±4.970 | 0.635±5.351 | .048 |
| **ACL force (N, N/BW)** |  |  |  |  |  |  |  |  |
| ACL* | 1469±84.3 | 1557±106.3^a^ | 1660±60.6^abc^ | .472 | 1486±62.9 | 1529±56.5^a^ | 1560±102.9 | .145 |
| Normalized ACL* | 2.036±0.330 | 2.147±0.377^a^ | 2.314±0.388^abc^ | .095 | 2.048±0.316 | 2.108±0.318^a^ | 2.140±0.353 | .014 |
| **Knee force (N, N/BW)** |  |  |  |  |  |  |  |  |
| Shear* | 398.4±98.8 | 576.2±154.3^ac^ | 864.2±204.7^abc^ | .613 | 348.8±62.5 | 504.8±115.5^a^ | 726.3±178.7^ab^ | .613 |
| Normalized shear* | 0.528±0.089 | 0.764±0.151^ac^ | 1.156±0.241^abc^ | .710 | 0.466±0.065 | 0.671±0.109^a^ | 0.971±0.216^ab^ | .691 |
| **Knee moment (Nm, Nm/(BW*HT))** |  |  |  |  |  |  |  |  |
| Sagittal | 83.41±24.50^c^ | 106.7±29.65^ac^ | 111.8±54.67^c^ | .100 | 49.04±17.69 | 48.77±28.35 | 48.60±24.03 | .000 |
| Normalized sagittal | 0.065±0.017^c^ | 0.084±0.025^ac^ | 0.086±0.041^c^ | .101 | 0.038±0.014 | 0.038±0.022 | 0.037±0.017 | .000 |
| Frontal* | 67.92±40.86^c^ | 160.2±89.49^ac^ | 209.0±93.69^abc^ | .376 | -57.52±34.15 | -66.15±17.66 | -97.82±48.72^ab^ | .202 |
| Normalized frontal* | 0.051±0.028^c^ | 0.122±0.066^ac^ | 0.160±0.067^abc^ | .407 | -0.046±0.028 | -0.052±0.015 | -0.078±0.043^ab^ | .181 |
| Transverse* | 25.64±15.70 | 11.31±21.34 | 14.34±31.22 | .069 | 11.16±10.07 | 16.66±8.62 | 37.04±20.19^ab^ | .408 |
| Normalized transverse* | 0.020±0.011 | 0.009±0.017 | 0.012±0.025 | .059 | 0.009±0.008 | 0.013±0.006 | 0.030±0.018^ab^ | .375 |
| **Muscle activation (%)** |  |  |  |  |  |  |  |  |
| Co-contraction index* | 0.546±0.188 | 0.290±0.167^a^ | 0.250±0.109^ac^ | .426 | 0.498±0.202 | 0.395±0.201 | 0.465±0.169 | .052 |

Note. Knee extension, adduction, and internal rotation are positive. ^*^ Indicates the significance in velocity × task interaction (*p*<.05), ^a^ Indicates the significance at 3 m/s (*p*<.017), ^b^ Indicates the significance at 4 m/s (*p*<.017), ^c^ Indicates the significance with the task (*p*<.017).
